# Immunooncology: Can the Right Chimeric Antigen Receptors T-Cell Design Be Made to Cure All Types of Cancers and Will It Be Covered?

**DOI:** 10.1155/2017/7513687

**Published:** 2017-01-23

**Authors:** Regina Au

**Affiliations:** BioMarketing Insight, Boston, MA, USA

## Abstract

Immunooncology (IO) is the buzz word today and it has everyone doing IO research. If we look back at the history of cancer treatment, the survival rate was measured in months which, according to oncologists, was a lot back then because the mortality rate in most cancers was 100%. However, most traditional chemotherapies were not well tolerated because they would kill both cancerous and healthy cells, which lead to major side effects such as loss of hair, nausea and vomiting, and risk of infection. Survival was better but not much better depending on the type of cancer and the patient's own genetic and physiological make-up. IO therapies target specific receptors on the cancer cells. However, with more advance technologies, the cost to develop these types of therapies increases significantly because the biology is more complex and it is more difficult to produce. Find out why these therapies are more complex and therefore more expensive. But the enhanced efficacy of these therapies does justify the cost.

## 1. Introduction

Scientists have tried to solve the targeting problem with IO therapies by utilizing the patient's own immune system to aid in recognizing and killing only cancer cells, rather than healthy cells, and keep the cancer cells at bay. Most recently, Chimeric Antigen Receptors T-cell (CAR-T) therapy is a cellular therapy that appears to be a game changer in cancer treatment. This review will cover (1) types of immunooncology therapies; (2) the different types of functioning T-cells; (3) the roadblocks to cellular therapies; (4) adaptive CAR-T therapy and the design of CAR-T-cells which are important; (5) the efficacy of CAR-T therapy in leukemia; (6) the efficacy of CAR-T in solid tumors; (7) the advantages of CAR-T therapy; (8) the side effect profile; (9) CAR-T therapy will be expensive; (10) CAR-T therapy does justify the cost; and (11) questions still facing the CAR-T field.

## 2. Types of Immunooncology Therapies

There are two categories of IO: (1) checkpoint therapies and (2) Adoptive Cell Transfer (ACT) therapies.

(1) Checkpoint therapies include cytokine therapy, therapeutic vaccine (dendritic cell vaccines), antibody drug conjugates, and tumor specific T-cell.

Checkpoint therapies currently on the market are Merck & Co.'s pembrolizumab (Keytruda®) [1] and Bristol Myers Squibb's (BMS) nivolumab (Opdivo®) [2] for specific types of cancers that have made significant inroads with some patients being cancer-free. Both Keytruda and Opdivo are human monoclonal antibodies that block the interaction between PD-1 and its ligands, PD-L1 and PD-L2, that inhibits the body's immune response, including antitumor immune response. BMS second monoclonal antibody ipilimumab (Yervoy®) [3] binds to CTLA-4 and blocks the interaction of CTLA-4 with its ligands CD80/CD86 that also inhibits T-cell activation and proliferation.

(2) Adoptive Cell Transfer (ACT) therapies, tumor infiltrating lymphocytes (TILs) from tumor mass that are excised, and gene transfer methods, Chimeric Antigen Receptors (CARs) T-cells and TCR (T-cell receptor) T-cells for blood, are included.

For the purpose of this review, we will focus on CAR-T therapy.

## 3. The Different Types of Functioning T-Cells

Our body has four basic types of functioning T-cells (Grupp 2014) [4]: (1) naive; (2) terminal effector (Te); (3) effector memory (Em); and (4) central memory (Cm). When there is no new infection present, the levels of naive cells are high and the rest are low. Once a bacteria or virus is introduced, there are high levels of T-Em, low levels of T-Cm, and no naive cells. When the T-cells are killing the bacteria or virus, there are high levels of T-Te and T-Em are low. These terminal effector cells, however, are subject to exhaustion and senescence and then they disappear. When the infection is cured, there are high levels of T-Cm that is activated when there is a reinfection.

## 4. The Roadblocks to Cellular Therapies

There are a number of roadblocks to cellular therapies [4], some of which scientists have figured out and others they still need to perfect.

*Problems*

*(1) Targeting*. CD19+ tumor cell and normal B cell both express CD19 where T-cells cannot recognize the tumor cells, getting T-cells to recognize only the cancer cells and not the normal cells.

*Solution*. T-cell recognition therapies are needed: CAR or TCR therapies; scientists have been developing these types of therapies and there are a number of CAR-T clinical trials worldwide.

*(2) Expansion Ex Vivo*. Making CAR-T-cells for each patient is complex and time consuming. We need a process where cells can expand significantly in cell culture or ex vivo.

*Solution*. We need to incorporate good manufacturing practice (GMP) cell culture approach in order for cells to proliferate significantly.

*(3) Expansion in Host*. Getting “programmed” T-cells to expand or proliferate significantly in the human body: in order to get an effector T-cell response, cells have to proliferate tremendously from a small number of precursor cells to a large number of effector cells and then convert to a memory response. This requires an enormous amount of T-cells.

*Solution*. We need to incorporate a significant amount of young T-cells that are not exhausted due to the expansion. This has not been proven yet.

*(4) Persistence*. We should get “programmed” T-cells to remain in the body for a long-term effect.

*Solution*. We need to use central memory T-cell (T-Cm) that will recognize the cancer cells for an unlimited period of time. This has not been proven yet.

*(5) Effector Cells and Target Ratio*. We must create more efficient effector memory T-cells (T-Em) with more cytotoxic T lymphocytes (CTL) activity.

*Solution*. We need proliferation or expansion of T-Em that will eventually convert into central memory T-cells (T-Cm), and it is the T-Cm cells that will activate and maintain CTL activity, when exposed to a bacteria or virus again. Efficiency and safety need to be demonstrated in Phase I clinical trials, not just safety, in order to demonstrate long-term efficacy in subsequent trials.

## 5. Adaptive CAR-T Therapy

**Figure 1:**
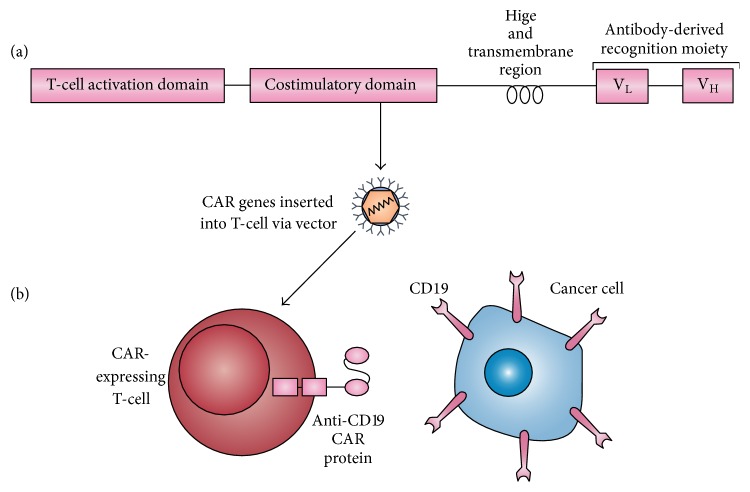
An anti-CD19 CAR-expressing T-cell recognizing a CD19+. Reprinted by permission from Macmillan Publishers Ltd. [34].

In ACT, more specifically CAR-T-cells, a CAR gene has two major components: (1) an external receptor that recognizes an antigen binding site on the cancer cells and (2) an internal component or signaling/expression that directs the T-cell to the cancer binding site and is inserted into a T-cell via a retrovirus or lentivirus vector. See Figure 1.

### 5.1. The Design of CAR-T-Cells Is Important

Scientists have been working on CAR-T-cells for over a decade and developed the first generation of CAR-T back in 1991 for HIV. This first generation of CD4/CD8z (CD4/CD8 T-cells) + CD3 *ζ*- (zeta) chain (to generate an activation signal in T lymphocytes) and CAR-T for HIV (CD4) using a retrovirus went into clinical trial in 1997; the CAR-T-cells persist now for 10 years (June 2015) [5].

The second generation of CAR-T uses a single chain fragment variable (scFv) or antibody fragment as the external component designed with the internal signaling CD28 or 4-1BB (CD137) + CD3 *ζ*-chain. Immunologists have found that they needed two (2) signals; signal 1 for activation and signal 2 for survival for T-cell proliferation (Looney 2013 [6], Maude et al. 2015 [7], and Sadelain et al. 2013 [8]). They looked at the power of dual signaling for proliferation or activation of T-cells. In one study, if you are using only signal 1, the anti-CD3 signal was low. If you are using only signal 2, the anti-CD28 signal was absent. Combining anti-CD3 and anti-CD28, the signal had at least a 1,000-fold increase (57K units). Scientists discovered that if you have signal 2 but not signal 1, it has no effect on T-cell. If you have signal 1 but no signal 2, there is inactivation (anergy) or deletion of T-cell (Hanada and Restifo 2013) [9].

**Figure 2:**
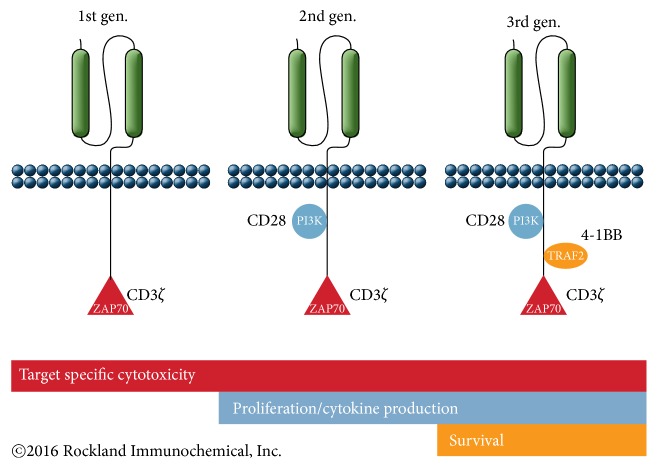
CAR-T designs. Source: Rockland Immunochemicals, Inc., http://www.rockland-inc.com/car-t-cell-therapy-services.aspx.

The costimulatory properties of second-generation Chimeric Antigen Receptors (CARs) determine the overall potency of adoptive transferred T-cells. But the combination of CD28-4-1BB and CD28-OX40 has demonstrated sustained activation of T-cells in animal models but remains to be evaluated in clinical trials (Almåsbak et al. 2016) [10]. Zhao and colleagues investigated seven (7) different CAR structures of CD28 and/or 4-1BB costimulation. They discovered that using the two signaling domains (CD28 and CD3*ζ*) configuration and the 4-1BB ligand provided the highest therapeutic efficacy, by providing balanced tumoricidal function and increased T-cell persistence which was accompanied by an elevated CD8/CD4 ratio and decreased exhaustion (Zhao et al. 2015) [11]. The costimulatory signals currently used in third-generation CAR-T-cells are CD28 and 4-1BB or Ox40 + CD3 *ζ*-chain. Scientists also believe that the microenvironment plays an important role in the immune system. See Figure 2 (NCI 2014) [12].

To make these cancer-fighting T-cells or CAR-T-cells, T-cells are first collected from the patient and then modified as in Figure 2 to recognize an antigen binding site on the cancer cells. It usually takes 2 and 1/2 to 3 weeks to insert the gene and grow cells. Once this is accomplished the modified T-cells are then infused back into the patient, or autologous therapy.

Once it is infused back into the patient these “programmed” T-cells can multiply and persist for a long time (“living drug”); they are capable of destroying any cells that have the target antigen.

This disruptive technology of modifying T-cells is similar to monoclonal antibody therapy, as it does use a fragment of an antibody, but has more potency and persistence, as living cells that can persist in the body, as opposed to antibodies (proteins) that are active for a limited time.

## 6. The Efficacy of CAR-T Therapy in Leukemia

In designing CD19 for Leukemia, three (3) different CAR-T therapies were compared from Memorial Sloan Kettering Cancer Center (MSKCC), the National Cancer Institute (NCI), and University of Pennsylvania, (UPenn) [5] (Davila et al. 2012) [13]; see Table 1. Professor Carl June emphasized that the design of the CAR-T is very important in addition to which vector you choose.

Viruses can transfer their genetic material to infected cells and then replicate, encapsulate, and package their genome to transfer to other noninfected cells. They were also found to incorporate and transmit genes of cellular origin making them an ideal tool to genetically modify cells [13] (Dufait et al. 2012) [14]. Retrovirus has been the most successful in human gene therapy in correcting genetic disorders. There are two basic types of retroviruses, simple, as in the Moloney mouse leukemia virus (MLV), and complex retroviruses (lentiviruses).

The difference between simple *γ*-retroviruses and a complex lentiviruses is that simple retrovirus vectors can only transduce cells during mitosis, while lentiviral vectors can transduce cells independently of their division status [14]. This characteristic makes lentiviral vectors ideal for gene therapy for highly differentiated cells.

For Acute Lymphocytic Leukemia (ALL), this is the type of efficacy data that has got everyone very excited about CAR-T therapy [5, 12] (UPenn [15], MSKCC [16], and NCI [17]). Even though the results for Chronic Lymphocytic Leukemia (CLL) were not as successful, it is a more complex disease to tackle and many companies are focusing on this area. In the UPenn study, the lentivirus probably contributed to the longer expression or persistence of CAR-T-cells. Due to these promising results, as of September 16, 2015, 77 CAR-T trials are being conducted around the world (48 in US, 8 in UK, and 20 in China) and China is predicted to outpace the US in clinical trials [5].

Emily Whitehead, the first pediatric patient with ALL treated with CAR-T therapy at the Children's Hospital of Philadelphia (CHOP), was a complete responder in 2012. She remains cancer-free for 4 years so far. When President Obama announced the Precision Medicine Initiative, Emily was invited to the White House in January of 2015 as a successful example of Precision Medicine or Personalized Medicine.

## 7. The Efficacy of CAR-T in Solid Tumors

CAR-T therapy has been successful combating circulating tumors such as ALL because the tumors are in the vicinity of the CAR-T-cells. However in solid tumors, the CAR-T-cells need a mechanism to hone in onto the cancer cells.

Checkpoint inhibitors or monoclonal antibodies have been used to treat prostate cancer due to its specificity in honing in on the tumor through prostate-specific membrane antigen (PSMA) and prostate stem cell antigen (PSCA). However, significant clinical efficacy has not been demonstrated. Surgery is generally the treatment for localized prostate cancer, but a high percentage of patients have a reoccurrence of tumors that progress to the lymph nodes and bone.

T-cells unlike antibodies have the ability to penetrate inflamed epithelial tissues, clonally expand, and generate memory cells producing a stronger antitumor activity thereby making modified CAR-T-cells directed at PSMA possibly a better treatment option. Hillerdal and Essand [18] and Abate-Daga et al. [19] both showed delayed tumor growth but not cure in mice treated with PSCA CAR-T-cells based on the 1G8 and Ha1-4.117 antibodies, respectively. Interestingly, Abate-Daga first developed a PSCA CAR-T to treat pancreatic cancer since PSCA is a glycoprotein overexpressed in early stages of malignancy in pancreatic cancer.

In prostate cancer that progresses to lymph node and bone metastases, T-cell infiltration is influenced by blood vessel quality and bone metastases have poor vessel quality with dysfunctional junctions [18]. The microenvironment or immunosuppressive environment creates many challenges in treating metastatic bone cancer. There are many factors that may enhance the CAR-T-cell efficacy in the tumor environment including using antiangiogenesis drugs for increased T-cell infiltration, targeting tumor stroma to improve antitumor effect, and depleting Treg as preconditioning, to TGF*β* blocking agent to reduce immunosupression and osteolysis [18]. But more studies are needed to confirm these hypotheses.

The first successful clinical trial using CAR-T therapy to treat solid tumors in pancreatic cancer was presented at the American Society of Clinical Oncology (ASCO) Annual Meeting in 2015. Six patients with refractory pancreatic cancers were treated: four patients showed progressive disease and two had stable disease (for 3.7 and 5.3 months) including one patient that resulted in the absence of some metastatic lesions (Castellino 2015) [20]. The investigators, from the University of Pennsylvania in Philadelphia, used specialized CAR-Tmeso (mesothelin + and a costimulatory molecule, 4-1BB) cell which was shown to hone in on the tumor sites of the patients. Mesothelin (MSLN) is a membrane-anchored protein normally seen in mesothelial cells and is overexpressed in all pancreatic cancer tissue according to investigator Gregory L. Beatty, M.D., Ph.D., assistant professor of medicine.

However, solid tumors in pancreatic cancer are uniquely challenging as they may have many different types of markers that have not yet been discovered. Researchers at the Perelman School of Medicine at Penn discovered a novel marker in a patient's tumor that did not possess any of the usual markers but had a specific change in protein glycosylation, a unique pattern of sugars on the cell surface of the protein (Cell Press 2016) [21]. In collaboration with other researchers, they developed a novel CAR-T-cells that expressed a monoclonal antibody called 5E5, which specifically recognizes the Tn glycan on the mucin 1 (MUC1) protein, a sugar modification. This marker is absent on normal cells but abundant on different types of cancer cells [21] (Posey Jr. et al. 2016) [22]. 5E5 modified CAR-T-cells were injected into mice with leukemia and pancreatic cancer that resulted in reduction of tumor growth and increased survival. All six mice with pancreatic cancer were alive at the end of the experiment.

## 8. The Advantages of CAR-T Therapy

There are a number of advantages to CAR-T therapy [4, 5, 7] (Brentjens 2015) [23]:

1. It uses human leukocyte antigen (HLA) restricted T-cell receptors, similar to a monoclonal antibody; it has very specific antigen recognition and therefore can have universal application.
2. It is active in both CD4 and CD8 T-cells.
3. It can target antigens on proteins, carbohydrates, and glycolipids.
4. It can rapidly produce tumor specific T-cells with GMP processes in 7–12 days.
5. It has minimal risk of autoimmunity or Graft versus Host Disease (GvHD).
6. It is a “living drug” that requires only a single infusion and persists for a unlimited period of time.
7. It destroys the cancer cell membrane in killing the tumor cell, and therefore, there is no cross reactivity unlike traditional chemotherapy.
8. CAR scFv or TCR (external) can reprogram specificity of T-cells for tumor target. Specificity is important to avoid toxicity.
9. CAR signaling domains can reprogram T-cell metabolism. This can enhance survival in tumor microenvironment and effector function.

The metabolism of CAR-T-cells plays an important role in the microenvironment, which had two phases: (1) resting to effector or activation (living off sugar) and then (2) effector to resting or memory cell (living off the mitochondrial) where memory cell will activate should there be a reinfection.

Therefore, choosing the appropriate signals can arm the T-cells better [5]:

1. CD28 domains: studies show that CD28 costimulation of human peripheral blood T-cells enhances expression of glucose transporters, glucose uptake, and glycolysis (Frauwirth et al., 2002) [24], the “Warburg” effect. After antigen encounter, T-cells shift to a glycolytic metabolism to sustain effector function (Sukumar et al. 2013) [25]. However, induction of high glycolytic activity in CD8^+^ T-cells severely compromises the ability of CD8^+^ T-cells to form long-term memory or decreased persistence.
2. 4-1BB domains enhance mitochondrial biogenesis and are associated with enhanced persistence. 4-1BB enhances primary CD8^+^ T-cell responses and the maintenance of memory CD8^+^ T-cells (Zhong et al. 2010) [26] associated with enhanced persistence.

## 9. The Side Effect Profile

One of the potentially lethal side effects with CAR-T therapy is cytokine release syndrome (CRS) which involves elevated levels of several cytokines including interleukin- (IL-) 6 and interferon *γ*. Clinical symptoms include fever, hypotension, respiratory insufficiency, and neurological changes such as delirium, global encephalopathy, aphasia, and seizure-like activities/seizure. This particular side effect was not evident in mice models and was presented only when it was infused into humans.

There were several cases of CRS at Children's Hospital of Philadelphia (CHOP) with significantly elevated levels of IL-6 which made the patients extremely ill. After a cytokine blockade failed, one 8 mg/kg dose of an IL-6 receptor antagonist, Tocilizumab, and the IL-6 levels returned to normal (Maude et al. 2015) [4, 7, 12]. IL-6 is a classic feedback loop mechanism possessing a network effect and one needs to interrupt multiple nodes or block the IL-6 mechanism to halt this toxicity.

It was also found that, by measuring the percentage of bone marrow blast (BMB), defined as disease burden, BMB correlates with the severity of CRS in children. Those with no disease burden are characterized as having BMB below 50%, and those with disease burden (yes) have greater than 50% BMB. Those who have a “yes” for disease burden have a greater likelihood and severity of CRS [4, 7]. It is more advantageous to deploy therapy in patients with a low burden of disease resulting in less toxicity. The more the BMB, the more severe the CRS. This could also be applied to adults as they have a mature immune system compared to children, who are still developing their immune system.

Other side effects can include the following [4, 7]:

1. Macrophage Activation Syndrome (MAS)/Hemophagocytic Lymphohistiocytosis (HLH) depicted by extraordinarily high ferritin levels (16K to 415K ng/mL).
2. Coagulopathy-elevated D-dimer and low fibrinogen.
3. Hepatosplenomegaly (HSM) and transaminitis and increased transaminases (AST, ALT) coupled with nonspecific hepatitis.
4. Moderate marrow hemophagocytosis.

## 10. CAR-T Therapy Will Be Expensive

People want to live longer with a better quality of life. To achieve this, scientists have gone into uncharted waters in understanding the etiology or mechanism of action of diseases, which is not an easy feat. In order to achieve this, drug development has got longer and longer and, therefore, more and more expensive. On average, according to a 2016 study published by Tuft's Center for the Study of Drug Development, it takes 11 years (range 10–15) to develop a drug from research to approval with a cost of $2.6 billion dollars (DiMasi et al. 2016) [27].

In the past, drug development was mostly focused on small molecule of chemical pathways that were well known, where a chemical reaction will occur in the same manner whether it is the first time or the 10^*n*^ time. Then the industry moved into biologics or large molecule where the biology is more complicated, and one cannot predict how a cell is going to react each time. Large molecule drug discovery, therefore, is inherently more expensive and scientists also had to figure out a way to get these large molecules to the right target.

Today, there is IO therapy, which is extremely complex compared to small or large molecule. Scientists understand how our immune system works, but not thoroughly enough to know how the immune system will react when one starts to manipulate the human immune system.

In order to administer CAR-T therapy, scientists had to figure out the following steps to manufacturing this therapy:

1. Depending on the type of cancer, design a CAR with the best external and internal signals (signal 1 and signal 2).
2. Design it with the best viral vector to transmit the gene to the T-cell and proliferate.
3. Get the CAR-T-cells to expand ex vivo in order to infuse it back to the patient.
4. Get the reprogrammed cells to expand in the host and persist for unlimited amount of time in order for the patient to remain cancer-free.
5. Collect T-cells from the patient.
6. Reprogram the patient's T-cell to CAR-T-cells.
7. Infuse it back into the patient.
8. It usually takes 2 1/2 to 3 weeks to insert the gene and grow cells.
9. Average time from screening to implantation into the patient with CAR-Tmeso was 41 days.

All these steps take an extraordinary amount of scientific knowledge, experimentation, and time for each individual. This is truly personalized medicine. CAR-T therapy is uncharted territory and no one knows whether this will work for every individual, even if one is using an individual's own immune system. In the comparison study of SKMCC, NCI, and UPenn for leukemia, the therapy worked well for ALL in a small number of patients, but not very well for CLL. The expression of CAR-T-cells only lasted for 30 days in the SKMCC and NCI which may account for why the response rate was poor for CLL. However, even with an expression rate of greater than 4 years in the UPenn study, the overall response rate was 57% for CLL versus 90% for ALL.

For patients who only achieved a partial response or is nonresponsive to the CAR-T therapy, doctors and scientists have to figure out why the patient did not have a complete response. It may mean going back to the drawing board in designing a different CAR, using a different viral vector, or using a different type of T-cell. This path adds on cost to the CAR-T therapy. Or, they may decide to either add another drug or go with a different class of agents.

In calculating the cost to produce this therapy, because the process described has to be done separately for each individual, it becomes very costly. Manufacturing cost only comes down when there is economy of scale, and with CAR-T therapy, there is no economy of scale since it is personalized to each individual. The cost of this therapy can only be determined by the biotech company that is actually developing the CAR-T therapy.

This is the dilemma. Society wants personalized medicine yet who is going to pay for the cost of personalized medicine? Insurance providers will not pay for CAR-T therapy because it is unproven by regulatory standards right now as well as their standards; the side effect profile is risky even though it can be remedied, and it is very expensive. Today, most insurance companies will only pay for the standard treatments and only when all therapies fail will the insurance company consider adoptive cellular therapy with special circumstances.

## 11. CAR-T Therapy Does Justify the Cost

If one can use their own immune system to fight cancer, this is ideal and the therapy would be a one dose cure as opposed to traditional treatments including checkpoint inhibitors, where the patient would take the drug/biologic for a specific period of time and hope the cancer is eradicated. Any inhibitor is only viable for a limited period of time compared to programmed T-cells, which could be expressed for an unlimited time period. In non-CAR-T therapy, the cancer could return and the same or different drug/biologic would have to be administered again similar to a maintenance therapy versus a cure, which is less expensive in the long run. And as each episode of a relapse occurs, the odds of survival are diminished significantly because the body gets weaker and the cancer gets smarter in terms of resistance.

But the real answer relies on the payer. The insurance company will not pay for a new therapy unless it is proven that CAR-T therapy works and is a cure, by their standards, not just FDA approval, it is safe, and it saves the insurance company money. But in order to determine this, health economic data must be collected for a determined length of time to demonstrate not only efficacy and safety, but also the fact that the therapy saves the company money compared to standard of care treatment, which many times are generic versions of the drug or biosimilar of a biologic.

If the insurance company will not cover the therapy, then the patient or family will have to pay for it. But most patients and families can not afford it and they will have to rely on the standard treatment and hope for the best.

## 12. Questions Still Facing the CAR-T Field

The development of CAR-T therapy is at the beginning of its era. There are still many questions facing the CAR field [4, 5]:

1. Does persistence correlate with outcome?
2. Is long-term persistence of CAR cells desired?
3. Which approaches give durable persistence of CAR-Ts?
4. What is the best vector to introduce the CAR: retroviral, lentiviral, or nonviral vectors?
5. Which is better: scFv or endodomain construction?
6. What is the optimal T-cell type and composition of the infused product?
7. How can checkpoint therapy and CAR-T therapy be combined?

One of the issues that contribute to compromised immune response is T-cell exhaustion and senescence and loss of CD8 and CD4 T-cell function, which occurs with chronic infections and cancer. The addition of a PD1 inhibitor, which is a checkpoint inhibitor, can rescue partially exhausted cell [6] and why scientists should investigate how checkpoint and CAR-T therapies can be used in combination.

In addition to questions facing the CAR field, there are clinical questions such as degree of disease burden and pre- and postinfusion therapy that can affect how well the CAR-T therapy works. Juno Therapeutics' CAR-T trial was halted when three patients died due to neurotoxicity, an adverse event well recognized in cases with CAR-T therapy but mild to moderate in a previous trial (Timmerman 2016) [28]. Juno believes that the deaths may have been related to the type of preconditioning therapy that patients got before receiving their reengineered T-cell infusions. Fludarabine (can cause neurologic affects) [29], a chemotherapy agent, was added to cyclophosphamide as the preconditioning regiment months after the trial started. Scientists believed that the depletion of certain blood cells would create a more favorable environment for the programmed infused T-cells to engraft and start attacking cancer. Later, with the elimination of Fludarabine from their protocol, Juno received the green light to restart the trial.

In order to bring the cost of this complex therapy down, scientists are looking at the possibility of allogenic (donor) therapy in lieu of autogenic therapy. Cellectis, a French biopharmaceutical company, has developed an allogenic CAR-T therapy, “UCAR-T” or universal CAR-T therapy. A pediatric patient with a refractory relapsed ALL was given UCAR-T in the UK after all previous treatment had failed in 2015. According to the CEO of Cellectis, at that time, the patient did not exhibit any adverse effects such as CRS and the UCAR-T-cells were still active after three months (Labiotech 2015) [30]. With these results, in June of 2016, Cellectis announced the enrollment of their first pediatric patient in a Phase 1 clinical trial for acute B lymphoblastic leukemia (B-ALL) at the University College of London (UCL) (Cellectis 2016) [31].

Kite Pharmaceutical is also pursuing the same path by partnering with the University of California Los Angeles (UCLA) for an allogenic T-cell therapies developed by Dr. Gay Crooks at UCLA, a type of artificial cell culture system that would support ex vivo differentiation of T-cells from pluripotent stem cells (Biopharm Drive 2016) [32].

## 13. Conclusion

It has been established that CAR-T therapy can work thus far as a cure in some patients. But the design of the CAR can be very complex and critical since the choice of costimulatory signals will determine whether or not an immune response is induced (CD28) or inhibited (CTLA 4 and PD1) [6]:

1. Inadequate costimulation can weaken host defenses leading to infection of cancer.
2. Inappropriate costimulation can lead to allergy, autoimmunity, and graft rejection.
3. Inadequate coinhibition leads to autoimmunity or autoinflammatory disease.
4. Inappropriate coinhibition leads to immunologic exhaustion.

It is a fine balance between the design of the CAR-T-cells, the microenvironment, and clinical influences such as disease burden or pretherapy in order for this therapy to work. Two main areas that warrant further research are the following.

*(1) Determining Why Some Patients Only Have a Partial or No Response*. T-cell exhaustion, senescence, and loss of CD8 and CD4 T-cell function due to chronic infection; inadequate amount of efficient effector memory T-cells (T-Em) converting to central memory cells which leads to increased cytotoxic T lymphocytes (CTL) activity; and lack of enough central memory cells for persistence are all roadblocks to cellular therapy that could explain why some patients have partial or no response. It could also be another mechanism that researchers will discover upon further research.

*(2) Designing a CAR-T Therapy That Is Specific to Each Type of Solid Tumors*. The complexity of treating solid tumors is twofold: (1) the T-cells have to hone in onto specific binding sites of the cancer cell and (2) T-cell infiltration can be hindered by poor microenvironment such as poor vessel quality and dysfunctional junction or toxic immunosuppressive environment as in prostate cancer. There are also a number of proteins that are overexpressed in many different types of solid tumors.

For pancreatic cancer, PSCA, Tn glycan on the mucin 1 (MUC1), and mesothelin are all overexpressed. Prostate and pancreatic cancer have the same PSCA that is overexpressed but PSCA is overexpressed in the premalignant stages of pancreatic cancer, and MSLN is overexpressed at later stages according to studies by Abate-Daga et al. [19]. Two different types of protein are overexpressed depending on the stage of the cancer, adding to the complexity in developing the right CAR-T to treat tumors. Castellino and his group [20] were the first to developed CAR-Tmeso cells to treat pancreatic cancer in mice.

Choosing or discovering the right target or targets for each individual in addition to knowing which protein to target at the appropriate stage of the cancer appears crucial in finding successful treatments for cancer. Many companies in the past have tried to use two, sometimes three, immunotherapies for pancreatic cancer, but have not produced favorable results since Gemcitabine (Gemzar) was approved in 1996 for pancreatic cancer. However, Gemzar only works in about 10% of the patients.

Apexian pharmaceuticals is taking a different approach in developing a drug that binds to APE1/Ref-1, a dual protein that is crucial in the development and growth of tumors in pancreatic cancer and is particularly dependent on APE1/Ref-1 [33]. In preliminary studies, it has shown to have activity in different types (breast, prostate, renal, head and neck, and colorectal) of cancers. If this drug comes to fruition, perhaps combination therapy with this drug and CAR-T for partial responders or nonresponders will result in more patients being and remaining cancer-free.

The issue of cost is always a topic at hand as to who is going to pay for these advance therapies. But it is already costing the healthcare system significant amount of money every time a drug does not work on a patient just because the other drugs are cheaper. Each failure adds cost to the system in keeping the patient in the hospital or returning to the emergency room due to complications as the cancer progressing further. A patient also should not have to suffer through failure after failure just because the other drugs are cheaper when one dose of a CAR-T therapy could have cured the patient. These new therapies should be covered by insurance. When allogenic CAR-T-cells are demonstrated to work as well as autogenic CAR-T-cells, the cost of therapy will definitely decrease significantly.

There has been many legislative orders from Obamacare to curb the rising cost of healthcare. But what some may not realize is that as people are living longer, they require more healthcare due to more comorbidities, which automatically increases the cost of healthcare. In order to prevent healthcare cost from spiraling out of control, the whole healthcare system has to change. Until we can change the mindset of everyone to foster preventive care, have patient's take responsibility of their own health, embrace the notion of treating the right patient with the right drug, with the freedom of advance technology being covered by insurance, and foster the expectation that not every disease needs to be treated with a machine gun when a pistol will achieve the same outcome, things will not change.

The advances in technology that scientists have made today are extraordinary, where cancer may be “cured” rather than in “remission.” The concept of cure would have been considered fiction 25 years ago. We have the technology. We should be able to use the technology and trust that people will use it appropriately.

## Figures and Tables

**Table 1:** CD-19 designs for leukemia.

	MSKCC	NCI	UPenn
Design	CD28-19-28z	CD28-FMC63-28Z	4-1BB-CD19-BB
Vector	Retrovirus	Retrovirus	Lentivirus
Expression	Approx. 30 days	Approx. 30 days	>4 years
CR in ALL	90%	80%	90%
CR in CLL	0/8		4/14
PR in CLL	0/8		4/14
OOR in CLL	0.00%		57%

CR: complete response, ALL: acute lymphocytic leukemia, CLL: chronic lymphocytic leukemia, PR: partial response, and ORR: overall response rate.
